# Differential mechanisms of action of the trace amines octopamine, synephrine and tyramine on the porcine coronary and mesenteric artery

**DOI:** 10.1038/s41598-019-46627-5

**Published:** 2019-07-29

**Authors:** Andy Hsien Wei Koh, Russ Chess-Williams, Anna Elizabeth Lohning

**Affiliations:** 1 Faculty of Health Sciences and Medicine, Bond University, 4229 Queensland, Australia; 2 Centre for Urology Research, Faculty of Health Sciences and Medicine, Bond University, 4229 Queensland, Australia

**Keywords:** Cardiovascular diseases, Cardiovascular biology

## Abstract

Trace amines such as *p-*tyramine, *p-*octopamine and *p-*synephrine are found in low concentrations in animals and plants. Consumption of pre-workout supplements containing these plant-derived amines has been associated with cardiovascular side effects. The aim of this study was to determine the mechanisms of action of these trace amines on porcine isolated coronary and mesenteric arteries. Noradrenaline caused contraction of mesenteric arteries and relaxation of coronary arteries. In both tissues, all three trace amines induced contractions with similar potencies and responses were unaffected by the β-adrenoceptor antagonist propranolol (1 µM), the nitric oxide synthase inhibitor L-NNA (100 µM), or the TAAR-1 antagonist, EPPTB (100 nM). However, the contractile responses of mesenteric arteries, but not coronary arteries, were significantly reduced by depletion of endogenous noradrenaline. Mesenteric responses to all three amines were abolished in the presence of prazosin (1 µM) whereas residual contractile responses remained in the coronary artery which were inhibited by a high concentration (100 µM) of EPPTB. The results suggest complex responses of the coronary artery to the trace amines, with activity at α_1_-adrenoceptors and potentially TAARs other than TAAR-1. In contrast the actions of the amines on the mesenteric artery appeared to involve indirect sympathomimetic actions and direct actions on α_1_-adrenoceptors.

## Introduction

**Figure 1:**
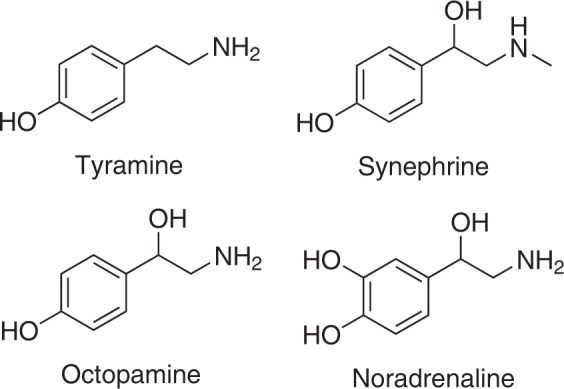
Chemical structures of the trace amines (tyramine, octopamine, and synephrine) and noradrenaline.

*p-*Tyramine (tyramine), *p-*octopamine (octopamine) and *p-*synephrine (synephrine) are substituted phenethylamines with a phenolic hydroxyl group in the *para*-position (Fig. 1). They are found in nanomolar concentrations in the mammalian nervous system and have thus been described as “trace amines”^1^. They also occur naturally in citrus plants such as *Citrus aurantium*^2^. Since 2004, *Citrus aurantium* extracts have been marketed as ergogenic and weight-loss aids, but there are limited studies to support this claim^3^. The safety of *Citrus aurantium*-listed supplements is still debated as adverse cardiovascular effects have been associated with their use^4^.

A number of actions of these trace amines on cardiovascular tissues has been reported. In some vascular tissues, tyramine has been shown to act as an indirectly acting sympathomimetic agent, promoting the release of endogenous noradrenaline^5^. Moreover, tyramine and octopamine cause nitric oxide dependant vasodilatory responses in pre-contracted rat mesenteric vascular beds^6^. Synephrine and octopamine are weak direct agonist effects at the α_1_-adrenoceptors of the isolated rat aorta^7–9^. In addition, octopamine and synephrine have been shown to exert weak direct β_1_-adrenoceptor agonist effects on isolated cardiac tissues and to activate cloned β_2_-adrenoceptors^10,11^. Thus, the amines can potentially exert cardiovascular effects via both direct or indirect mechanisms, but the extent of these actions in different vessels of the same species has not been explored.

Recently it has been recognised that these amines may exhibit some activity mediated via trace amine-associated receptors (TAARs)^12,13^. Six subtypes of this receptor have been identified, but knowledge of their distribution and functions is limited. All three amines have been shown to activate human TAAR-1 expressed in cloned cells and increase cAMP levels, with tyramine being the most potent drug^12–14^. It has been hypothesised that tyramine and octopamine acts directly on TAARs to cause vasoconstriction in endothelium-denuded rat aortas^15,16^ and pig coronary arteries^17^, but no selective antagonists were available to investigate this hypothesis. The presence of specific tyramine receptors had previously been proposed to explain rat aortic responses to synephrine that were resistant to conventional receptor antagonists^8^. Thus, there is some indirect evidence to suggest that tyramine and synephrine may induce some vascular responses via specific receptors.

The aim of the present study was to investigate the actions and mechanisms of action of the trace amines on the mesenteric and coronary artery using a porcine model. The mesenteric artery is densely innervated by the sympathetic nerves, and is a major regulator of blood flow to the gastrointestinal system during physiological stress^18^. Blood flow to the intestine can be drastically reduced (>80%) during exercise which results in the shunting of blood to the skeletal and cardiac muscle^19^. In contrast, during physiological stress the adrenergic nervous system ensures an increased blood flow to cardiac muscle via the coronary circulation^20,21^. Thus, in this study, the relative potencies of the trace amines tyramine, octopamine and synephrine were investigated on the vascular tone of mesenteric artery and the coronary artery and the mechanisms of action for each amine in the two functionally very different arteries determined. The role of TAAR-1 in responses was also examined using EPPTB (RO-5212773), a recently developed selective antagonist for TAAR-1^22^.

## Results

### Responses of the porcine mesenteric artery

**Figure 2:**
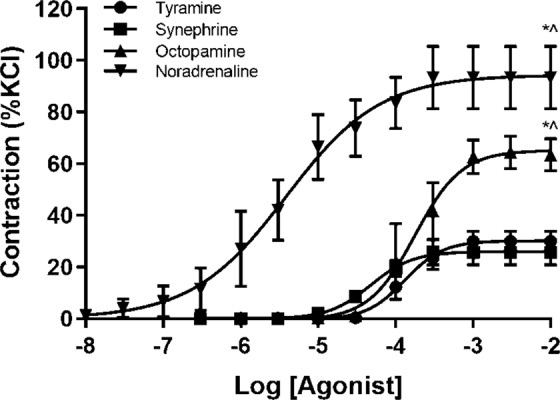
Initial cumulative-dose responses of trace amines and noradrenaline on isolated mesenteric artery. The amines shown are tyramine (● n = 6), synephrine (■, n = 6) and octopamine (▲, n = 6). The data are presented as percent of contraction to 60 mM potassium chloride (%KCl). Welch corrected unpaired t-test, *, p < 0.05 vs. tyramine; ^, p < 0.05 vs. synephrine.

All three trace amines produced concentration-dependent contractions of the porcine arterial rings (Fig. 2). Tyramine and synephrine produced similar maximum responses, whilst those to octopamine were significantly greater (unpaired Welch’s *t*-test, *p* < 0.05). All three trace amines had similar potencies that were not significantly different (*p*EC50 ranging from 3.25–3.91). The endogenous amine, noradrenaline produced greater contractions than the three trace amines and was also the most potent drug tested (Fig. 2, Table 1).

**Table 1:** Effects of antagonists on the maximum responses and potency values of p-synephrine, octopamine, tyramine, noradrenaline and phenylephrine in porcine mesenteric arteries. Paired Student’s t-test *p < 0.05 vs control.

Trace amine and interventions	Controls		Treatment		Sample number (n)
	Max contraction (%KCl)	Potency (pEC50)	Max contraction (%KCl)	Potency (pEC50)	
**Tyramine**					
Tyramine pre-treatment (3 mM)	30.0 ± 3.9	3.91 ± 0.14	2.9 ± 0.7*	2.87 ± 0.06*	6
Propranolol (1 µM)	2.9 ± 0.7	2.49 ± 0.19	2.9 ± 0.6	2.88 ± 0.27	6
Prazosin (1 µM)	2.9 ± 0.7	2.49 ± 0.19	Abolished*	Abolished*	6
L-NNA (100 µM)	4.2 ± 2.7	2.50 ± 0.23	6.5 ± 3.0	2.81 ± 0.56	4
**Synephrine**					
Tyramine pre-treatment (3 mM)	25.6 ± 4.8	3.81 ± 0.12	10.9 ± 2.6*	3.76 ± 0.26	6
Propranolol (1 µM)	12.5 ± 2.9	3.90 ± 0.51	16.1 ± 3.8	3.45 ± 0.41	6
Prazosin (1 µM)	9.6 ± 2.6	3.81 ± 0.56	Abolished*	Abolished*	5
L-NNA (100 µM)	19.2 ± 10.1	4.06 ± 0.68	17.4 ± 9.8	2.29 ± 1.19	4
**Octopamine**					
Tyramine pre-treatment (3 mM)	63.4 ± 6.3	3.25 ± 0.12	19.9 ± 2.3*	3.60 ± 0.21	5
Propranolol (1 µM)	17.1 ± 3.6	3.80 ± 0.44	15.4 ± 4.4	3.46 ± 0.52	5
Prazosin (1 µM)	14.0 ± 2.8	3.80 ± 0.45	Abolished*	Abolished*	6
L-NNA (100 µM)	16.7 ± 9.3	3.80 ± 0.44	12.8 ± 6.0	3.55 ± 0.41	6
**Noradrenaline**					
Tyramine pre-treatment (3 mM)	85.7 ± 12.2	5.42 ± 0.21	85.9 ± 6.5	5.22 ± 0.13	5
Propranolol (1 µM)	81.9 ± 5.9	5.92 ± 0.14	91.4 ± 4.4	5.65 ± 0.09	5
Prazosin (1 µM)	87.7 ± 4.7	5.92 ± 0.14	81.9 ± 5.2	5.10 ± 0.08*	5

### Responses of the porcine coronary artery

**Figure 3:**
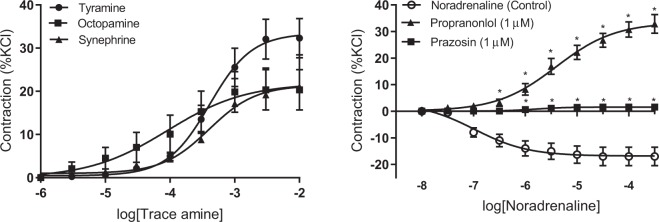
(Left) Concentration-response curves of the porcine coronary artery to tyramine, octopamine and synephrine (n = 6–8). (Right) Concentration-response curves to noradrenaline in the absence of antagonists and in the presence of the β-adrenoceptor antagonist propranolol (1 M) and in the combined presence of propranolol (1 µM) and the α1-adrenoceptor antagonist prazosin (1 µM). Responses are expressed as a percentage of the contractile response to potassium chloride (60 mM). Student’s paired parametric t-test *p < 0.05 vs. the relaxation before addition of antagonists.

All three trace amines produced concentration-dependent contractions of porcine coronary arterial rings (Fig. 3). Octopamine and synephrine produced similar maximum responses, whilst those to tyramine were greater, the difference between tyramine and octopamine being statistically significant (*p* < 0.05). The potencies of all three amines were similar and ranged from 3.30–3.88 (Table 2). The endogenous amine noradrenaline failed to produce contraction and only relaxations were observed (Fig. 3). These relaxations were converted to contractions in the presence of the β-adrenoceptor antagonist propranolol (1 µM). The maximum contractile responses to noradrenaline (33.4 ± 2.7% of the response to potassium) were significantly greater than those to octopamine (20.3 ± 4.6%, *p* < 0.05) and synephrine (21.1 ± 7.3%, *p* < 0.05), but not tyramine (32.3 ± 4.5%). In the presence of both propranolol (1 µM) and the α_1_-adrenoceptor antagonist prazosin (1 µM) responses to noradrenaline were abolished completely.

**Table 2:** Effects of antagonists on the maximum responses and potency values of tyramine, octopamine, synephrine and noradrenaline in porcine coronary arteries. Paired parametric Student’s t-test *vs. controls p < 0.05. **vs. controls p < 0.01.

Trace amine and interventions	Controls		Treatment		Sample number (n)
	Max contraction (%KCl)	Potency (pEC50)	Max contraction (%KCl)	Potency (pEC50)	
**Tyramine**					
Tyramine pre-treatment (3 mM)	32.3 ± 4.5	3.30 ± 0.13	34.12 ± 6.5	3.37 ± 0.12	8
Propranolol (1 µM)	24.1 ± 3.8	3.27 ± 0.12	22.3 ± 2.9	3.23 ± 0.11	5
Prazosin (1 µM)	39.3 ± 7.5	3.51 ± 0.17	24.3 ± 5.9**	3.26 ± 0.21	6
EPPTB (100 nM)	22.8 ± 7.5	3.03 ± 0.31	31.4 ± 6.7	2.94 ± 0.14	6
EPPTB (100 µM)	13.4 ± 2.1	3.13 ± 0.09	4.0 ± 1.1**	3.10 ± 0.18	7
L-NNA (100 µM)	34.6 ± 4.5	3.42 ± 0.20	41.6 ± 4.1	3.27 ± 0.14	6
**Octopamine**					
Tyramine pre-treatment (3 mM)	20.3 ± 4.6	4.04 ± 0.43	13.7 ± 3.9	3.58 ± 0.29	6
Propranolol (1 µM)	12.6 ± 4.5	3.85 ± 0.68	20.7 ± 6.3	3.35 ± 0.34	5
Prazosin (1 µM)	13.7 ± 3.9	3.55 ± 0.27	5.9 ± 2.6*	3.15 ± 0.24*	6
EPPTB (100 nM)	6.5 ± 2.0	3.31 ± 0.88	8.1 ± 2.9	2.94 ± 0.25	5
EPPTB (100 µM)	16.5 ± 2.9	3.43 ± 0.18	7.8 ± 2.5*	3.04 ± 0.20	6
L-NNA (100 µM)	18.8 ± 6.0	3.13 ± 0.49	17.6 ± 1.5	3.27 ± 0.13	5
**Synephrine**					
Tyramine pre-treatment (3 mM)	21.1 ± 7.3	3.50 ± 0.26	13.1 ± 3.4	3.55 ± 0.19	6
Propranolol (1 µM)	10.5 ± 3.0	3.74 ± 0.44	16.9 ± 0.3	2.59 ± 0.29	7
Prazosin (1 µM)	22.5 ± 6.7	3.21 ± 0.80	6.0 ± 2.1*	3.70 ± 0.35	6
EPPTB (100 nM)	25.1 ± 7.2	3.42 ± 0.43	27.0 ± 5.1	3.54 ± 0.33	6
EPPTB (100 µM)	12.8 ± 4.3	3.82 ± 0.42	3.9 ± 1.3*	3.83 ± 0.36	8
L-NNA (100 µM)	34.4 ± 6.5	3.29 ± 0.28	24.8 ± 4.5	3.60 ± 0.31	6
**Noradrenaline**					
Propranolol (1 µM)	−16.9 ± 1.5	6.98 ± 0.48	33.4 ± 2.7**	5.40 ± 0.15*	6
Propranolol + Prazosin (1 µM)	−16.9 ± 1.5	6.98 ± 0.48	4.0 ± 1.5**	5.29 ± 0.65*	6

### The role of endogenous noradrenaline release in responses in mesenteric artery

**Figure 4:**
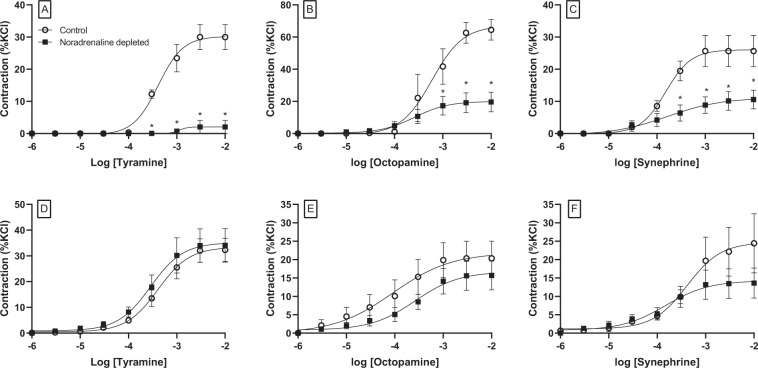
Concentration-response curves to tyramine (**A,D**), octopamine (**B,E**) and synephrine (**C,F**) in control tissues (○), mesenteric (**A–C**) and coronary arteries (**D–F**) previously depleted of neuronal NA (▪) using prolonged contact (60 mins) with a high concentration (3 mM) of tyramine. Data are means ± sem values from 6 to 24 experiments, expressed as a percentage of the contractile response to potassium chloride (60 mM). Student’s paired parametric t-test *p < 0.05 vs. control.

In mesenteric arteries, tyramine pre-treatment for 30 minutes nearly abolished subsequent responses to tyramine with a > 90% reduction in maximal contraction and a rightward shift of curves (*p*EC50 value 2.87 ± 0.06, *n* = *6; Student’s t-test p* < 0.05) (Fig. 4). In tissues depleted of endogenous noradrenaline, the maximal contractions to synephrine were reduced by 58% and maximal responses to octopamine were halved. However, the 30-minute tyramine pre-treatment did not affect the potencies (*p*EC50 values) of either synephrine or octopamine (Fig. 4). The contractile responses to exogenous noradrenaline were not significantly affected by tyramine pre-treatment (Table 1).

To examine whether responses to any of the trace amines involved the release of nitric oxide, tissues were incubated with the nitric oxide synthase inhibitor, L-NNA (100 µM). None of the responses to the trace amines was affected by the removal of nitric oxide (Table 1).

### The role of endogenous noradrenaline release in responses in coronary artery

Tyramine pre-treatment did not affect the potencies or maximal contractions to either tyramine itself or synephrine (Fig. 4, Table 2). Noradrenaline depletion did appear to reduce responses to octopamine by about 50%, but the change was not statistically significant (Fig. 4). None of the responses to the amines was affected by the removal of nitric oxide with L-NNA (Table 2).

### Role of direct adrenoceptor stimulation in trace amine-induced vasoconstriction in mesenteric artery

**Figure 5:**
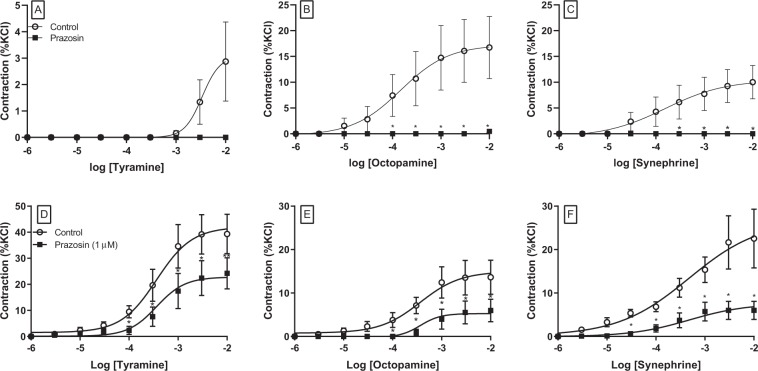
Concentration-response curves to trace amines in the absence (○) and presence of prazosin (1 µM, ■) in NA-depleted mesenteric arteries (**A–C**); and NA-depleted coronary arterial rings (**D–F**). Data are means ± sem (n = 5–7) values expressed as a percentage of the contractile response to potassium chloride (60 mM). Student’s paired parametric t-test *p < 0.05 vs control.

In noradrenaline-depleted mesenteric artery rings the presence of the α_1_-adrenoceptor antagonist prazosin (1 µM) abolished the responses to all three trace amines (Fig. 5, Table 1). Prazosin caused a rightward shift of concentration-response curves to noradrenaline (P < 0.05) without affecting maximum responses (Table 1). Neither maximum contractile responses nor potencies for any of the trace amines or noradrenaline were changed in the presence of propranolol (1 µM) (Table 1).

### Role of direct adrenoceptor stimulation in trace amine-induced vasoconstriction in coronary artery

In noradrenaline-depleted coronary artery rings the presence of prazosin (1 µM) reduced maximum contractions produced by tyramine, octopamine and synephrine by about half without significantly affecting agonist potency (Fig. 5, Table 2). Contractile responses to synephrine were the most significantly affected by prazosin, but responses were again not completely abolished. Neither maximum contractile responses nor potencies for any of the amines changed significantly in the presence of propranolol (1 µM) (Table 2).

### Role of Trace amine-associated receptor 1 (TAAR-1) in trace amine-induced vasoconstriction of coronary artery

**Figure 6:**
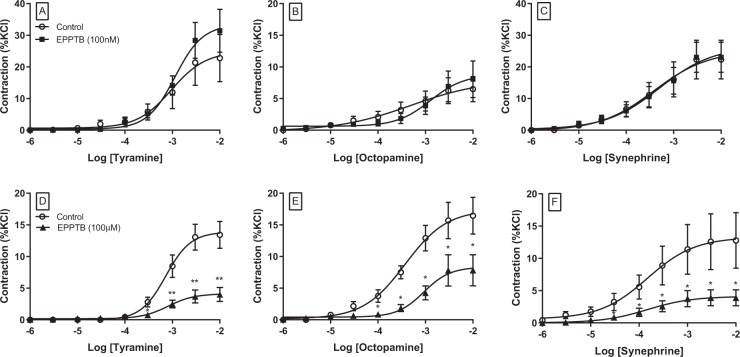
Concentration-response curves to tyramine (**A,D**), octopamine (**B,E**) and synephrine (**C,F**) in the absence (○) and presence of the TAAR-1 antagonist, EPPTB at 100 nM (■;**A–C**) or 100 µM (▲;**D–F**) in porcine coronary arterial rings. Data are means ± sem from 5 to 8 separate experiments, expressed as a percentage of the contractile response to potassium chloride (60 mM). Student’s paired parametric t-test *p < 0.05 vs. control, **p < 0.01 vs. control.

In noradrenaline-depleted tissues,the possible involvement of the TAAR-1 receptor in mediating responses was investigated using the selective TAAR-1 antagonist EPPTB (Fig. 6, Table 2). The effects of this antagonist were dependent on the concentration of the antagonist. At the lower concentration (100 nM), EPPTB did not reduce responses to any of the trace amines, and for tyramine, responses were greater in the presence of this antagonist, although the change was not statistically significant.

The higher concentration of EPPTB (100 µM) did not affect *p*EC50 values for any of the amines but reduced maximum responses to both tyramine and synephrine by 70%, whilst responses to octopamine were reduced by 53% (Fig. 6, Table 2).

## Discussion

All three trace amines contracted the mesenteric artery but were weaker and less potent than the biogenic amine noradrenaline. The potency order for the amines was noradrenaline > tyramine = synephrine = octopamine where the differences between the three trace amines were not significantly different. Previously vasoconstrictor effects of tyramine on canine mesenteric arteries^23,24^ and octopamine on rat mesenteric arterioles^25^ have been observed, but the vascular effects of synephrine are unclear. Huang *et al*. (1995) reported that synephrine was incapable of producing contractions of isolated rat mesenteric arteries^26^, but in our study synephrine produced concentration-dependent vasoconstrictions of the porcine mesenteric artery, suggesting that species differences may occur. In the same study by Huang and colleagues, a *Citrus aurantium* extract caused dose-dependent vasocontractions of the rat mesenteric artery with a maximum value of 27 ± 3% (% 60 mM KCl), a value similar to the maximum contractions observed in the responses to synephrine in this current study. In contrast to our experiments on isolated mesenteric arteries, Anwar and colleagues showed a vasodilatory response in perfused rat mesenteric beds^6^. The discrepancy between the reports may be a result of differences in perivascular nerve distribution, as there is a greater density in non-adrenergic non-cholinergic nerves 2^nd^ and 3^rd^ branches of mesenteric arteries^27^. Thus differences between the Anwar study and our study may be explained by differences in species (rat vs pig) of type of vessel examined (perfused vascular bed vs larger artery).

On the coronary artery, all three trace amines were partial agonists and contracted the tissues with potencies similar to those seen on the mesenteric artery (ie. pEC50 range 3.3–4.0). The observations for tyramine were consistent with previous studies on the porcine coronary artery^17^ and the rat aorta^8,9,15^. However, unlike the mesenteric artery, the left anterior coronary artery contains a high proportion of β_2_-adrenoceptors and relatively few α_1_-adrenoceptors^28^ and noradrenaline administered to control arteries caused vasorelaxation, which would be consistent with predominant β_2_-adrenoceptor activation. In the presence of the β-adrenoceptor antagonist, propranolol, the responses to noradrenaline were reversed to vasoconstriction. The constriction of the coronary smooth muscle in response to noradrenaline was caused by α_1_-adrenoceptors as evidenced by the abolition of responses in the presence of the α_1_-adrenoceptor antagonist prazosin. In contrast to noradrenaline, propranolol did not affect the responses to the trace amines, and even in the presence of prazosin, these amines could produce coronary artery vasoconstriction suggesting a non-adrenoceptor mechanism of action.

To further investigate the mechanisms involved in the vasoconstriction induced by the trace amines, the contribution of indirect actions and direct adrenoceptor activity were examined. The inhibition of NO synthase did not significantly affect responses to any of the amines indicating they did not stimulate release of NO. However, depletion of sympathetic stores of noradrenaline did have an effect and has been shown to greatly reduce the responses of tissues to indirectly acting sympathomimetic drugs^29,30^. Tyramine was first described as an indirectly acting sympathomimetic drug by Burn and Rand (1958), who showed that the depletion of endogenous noradrenaline stores, completely abolished the effects of tyramine but not directly acting amines^31^.

The mesenteric vasculature contains a dense neuronal network that would redirect blood flow away from the intestine during sympathetic nervous system activation^18^. The incubation of porcine mesenteric arteries with a high concentration (3 mM) of tyramine for 30 minutes almost abolished subsequent responses of tissues to tyramine, indicating that stores of noradrenaline were depleted. Responses to exogenous noradrenaline were not affected by prior depletion of noradrenaline stores, demonstrating direct activation of α-adrenoceptors by noradrenaline caused vasoconstriction. In contrast, the responses to octopamine and synephrine were halved in noradrenaline-depleted tissues, suggesting that, like tyramine, the release of endogenous noradrenaline from sympathetic nerves contributes, at least partially, to the responses of these mesenteric vessels to these trace amines.

Different results were obtained in the coronary arteries where the three trace amines continued to elicit significant responses after incubations to deplete endogenous noradrenaline stores. Responses to tyramine were almost identical in control and noradrenaline-depletion tissues, however responses to octopamine and synephrine appeared to be reduced slightly but the changes were small and not statistically significant. These results suggest that in the coronary artery, unlike the mesenteric artery, the release of endogenous noradrenaline from sympathetic nerves does not contribute significantly to the responses of coronary vessels to trace amines. This conclusion was further supported by the finding that the response of the coronary artery to noradrenaline in the absence of antagonists was relaxation, making it impossible for the release of endogenous noradrenaline to cause the contractile responses to the trace amines.

In mesenteric arteries the small contractile responses to the trace amines remaining after noradrenaline depletion were not affected by the β-adrenoceptor antagonist, propranolol, but were abolished in the presence of prazosin. This indicates that the amines lacked significant effects on vascular β-adrenoceptors in this tissue and that the residual contractile effects after noradrenaline depletion were mediated via α_1_-adrenoceptor stimulation. It has previously been reported that responses of the coronary artery to the trace amines, tyramine and β-phenylethylamine (β-PEA) were not affected by either prazosin or propranolol^17^, suggesting a lack of adrenoceptor involvement in responses. However, the coronary responses to synephrine and octopamine were not explored. In the present study, the maximum contractions of coronary arteries to all three trace amines were significantly reduced by prazosin indicating a weak partial agonist action at the α_1_-adrenoceptors of this tissue. These results are consistent with previous data using rat aorta where octopamine and synephrine activated α_1_-adrenoceptors to cause contraction^7,9,15^. However, the residual vasoconstrictions to these three amines in the presence of prazosin suggests the involvement of another non-adrenergic receptor. Other monoamines such as 5-HT, histamine and dopamine are known to cause constriction of pig coronary arteries, but blockade of their respective receptors does not affect responses to tyramine or β-PEA^17^. This prompted the authors to propose that these amines were acting on TAARs although an antagonist was not available at the time to test this hypothesis.

The physiological effects of trace amine-associated receptors were first identified in the process of olfaction, but these receptors have since been identified in tissues throughout the body. The TAAR-1 subtype is unusual as it is not involved in olfaction but is involved in neuromodulation and is therefore a potential target for drug development in the treatment of psychiatric and neurodegenerative disorders^32^. Stimulation of TAAR-1 causes activation of the G-protein alpha subunit (Gs) and subsequent increase in cellular cAMP concentration^12^. The selective competitive antagonist for TAAR-1, EPPTB (RO-5212773) was first described by^33^. It has a high affinity for the mouse TAAR-1 (Ki = 0.9 nM) and was employed in the present study at a concentration of 100 nM. The maximum responses to tyramine, but not octopamine or synephrine, appeared to be increased in the presence of 100 nM EPPTB although the effect was not statistically significant. Had tyramine activated TAAR-1 receptors enhanced responses would be expected since the receptor normally inhibits contraction. This would fit with the known activation of adenylyl cyclase following activation of this receptor and the inhibition of contraction by intracellular cAMP. It has been reported that tyramine is more potent at TAAR-1 than octopamine or synephrine^13,14^. However, the effects of EPPTB are known to be species-dependent^33^ since the drug exhibited a high affinity for the mouse variant of TAAR-1 (Ki = 0.9 nM) but a much lower affinity for the rat (Ki = 942 nM) and human TAAR-1 (Ki > 5 µM). The affinity of EPPTB at the porcine receptor is unknown, but we examined a higher concentration of the antagonist that would block responses mediated via the low-affinity variant of this receptor. In the presence of the higher concentration of EPPTB (100 µM), contractile responses to all three trace amines were reduced by more than half. Whether this represents an action of these trace amines at the low affinity TAAR-1 or a non-selective action at other TAAR receptors at higher drug concentrations is not known. Unfortunately, further studies will have to wait until selective drugs at other TAAR subtypes have been developed.

## Conclusion

This study has shown that the trace amines, (tyramine, octopamine and synephrine) can induce vasoconstriction by a variety of complex mechanisms including direct α-adrenoceptor activation, indirect actions via release of endogenous noradrenaline and actions at another receptor yet to be identified, with the mechanism depending on the blood vessel. On the mesenteric artery, the trace amines induced contractions via an indirect sympathomimetic action and also a direct α_1_-adrenoceptor agonist mechanism. In the coronary artery, the amines appeared to have almost no indirect sympathomimetic actions but do elicit contractions via weak direct agonist actions on α_1_-adrenoceptors and possibly an action on TAAR receptors other than the TAAR-1 subtype.

## Methods and Materials

### Tissue preparation

Hearts and gastrointestinal tracts from 5-month-old female pigs were obtained from the local abattoir and transported in ice-cold Krebs-bicarbonate solution to the laboratory. The left anterior descending coronary artery or the inferior mesenteric artery were isolated and 3 mm length rings were suspended between stainless-steel hooks and stationary supports in 8 mL organ bath (EZ-baths, Global Towns, CA) containing Krebs-bicarbonate solution (composition in mM: NaCl 118, NaHCO_3_ 25, glucose 11.7, MgSO_4_ 2.4, KH_2_PO_4_ 1.2, KCl 1.2, CaCl_2_ 2.5) maintained at 37 °C and continuously oxygenated with 5% CO_2_ in oxygen. The rings were mounted under a resting tension of 5 g and the tension developed by the circular smooth muscle in response to the addition of drugs was measured using isometric force transducers coupled to a PowerLab computer system (AD Instruments, Castle Hill, Australia).

### Experimental protocol

After equilibrating the tissues for 30 minutes, cumulative concentration-response curves to either noradrenaline, phenylephrine, tyramine, synephrine or octopamine were generated. After washout, responses to the amines were repeated and responses to KCl (60 mM) were obtained at the end of the experiment. Responses were expressed as a percentage of the response to 60 mM KCl.

To investigate the possible indirect sympathomimetic action of these amines; the tissues were incubated with a high concentration of tyramine (3 mM) for 30 minutes to deplete pre-synaptic noradrenaline (NA) stores. After washout, responses to noradrenaline, tyramine, synephrine or octopamine were again obtained.

To investigate the mechanisms involved in the functional actions of these amines; α_1_- or β-adrenoceptor antagonists (prazosin or propranolol respectively; both 1 µM), trace amine-associated receptor 1 antagonist (EPPTB; 100 nM and 100 µM) or the nitric oxide synthase inhibitor, L-Nω-Nitroarginine (L-NNA; 100 µM) were added 30 minutes before the addition of agonists. Control experiments without the addition of the antagonist were also performed, but repeated curves to the amines were not significantly different and correction of the second test curve in the presence of the antagonist was not required.

### Statistical analysis

Concentration-response curves were analysed using PRISM 8 (GraphPad Software, San Diego, USA). The data are represented as mean ± s.e. mean with *n* indicating the number of animals from which arterial rings were obtained. Comparison of the different concentration-response curves was performed using paired Student’s t-test when comparing 2 normally distributed groups (e.g. pEC50 in the absence v.s. presence of antagonists), or a Welch corrected unpaired Student’s *t*-test applied to the normalised responses which may have unequal variances. Statistical significance was demonstrated by a *p*-value of less than 0.05. Potency was expressed as the pEC50 value (-log EC50, which was the molar concentration producing a response 50% of the maximum effect).

### Drug and chemicals

(+)Noradrenaline-bitartrate, (±)*p*-synephrine, (±)*p*-octopamine hydrochloride, (±)*p*-tyramine, (+)-phenylephrine hydrochloride, prazosin hydrochloride, propranolol hydrochloride, EPPTB (RO-5212773, N-(3-Ethoxyphenyl)-4-(1-pyrrolidinyl)-3-(trifluoromethyl)benzamide) and Nω- nitro-L-arginine (L-NNA) were obtained from Sigma-Aldrich (Sydney, Australia). All drugs except RO-5212773 were dissolved in distilled water and then diluted with Krebs-bicarbonate solution. RO-5212773 was dissolved in 1% dimethyl sulfoxide (DMSO) prior to dilution with Krebs-bicarbonate solution.
